# Diagnostic prediction of ulcerative colitis using simplified endoscopic features and blood count-derived indices: a multicenter study

**DOI:** 10.3389/fmed.2026.1991253

**Published:** 2026-10-26

**Authors:** Yunlong Sun, Dongyu Cheng, Hairong Huang, Dongping Lai, Ri'an Xu, Dadong Wang, Hao Li, Linyan Shen, Tao Zhang

**Affiliations:** 1 Guangxi University of Chinese Medicine, Nanning, Guangxi, China; 2 Department of Gastroenterology, Minzu Hospital of Guangxi Zhuang Autonomous Region, Nanning, Guangxi, China; 3 Department of Gastroenterology, Guilin Hospital of Integrated Traditional Chinese and Western Medicine, Guilin, Guangxi, China; 4 Department of Gastroenterology, The Second Affiliated Hospital of Guangxi University of Chinese Medicine, Nanning, Guangxi, China; 5 Department of Gastroenterology, Guidong People’s Hospital of Guangxi Zhuang Autonomous Region, Wuzhou, Guangxi, China; 6 Department of Gastroenterology, Affiliated Wuxi Hospital of Nanjing University of Chinese Medicine, Wuxi, Jiangsu, China

**Keywords:** diagnostic prediction model, differential diagnosis, fecal calprotectin, mucosal granularity, systemic immune-inflammation index, ulcerative colitis

## Abstract

**Introduction:**

Fecal calprotectin (FC) results may be unavailable during assessment of inflammatory or ulcerative intestinal lesions. We assessed FC documentation and developed a diagnostic prediction model using endoscopic features and blood-derived inflammatory indices for ulcerative colitis (UC).

**Methods:**

We retrospectively included 655 patients from four centers. Four prespecified logistic models were compared in complete cases (*N* = 455; 103 UC cases). Performance included discrimination, Brier score, calibration, and decision curves. Internal validation used 1,000 bootstrap resamples; transportability was assessed by leave-one-center-out cross-validation. LASSO-based analyses were exploratory; multiple imputation also assessed the robustness of the four primary models.

**Results:**

FC was documented in 6 patients (0.9%). A model combining mucosal granularity, mucosal edema, neutrophil-to-lymphocyte ratio, and systemic immune-inflammation index had an apparent area under the receiver operating characteristic curve (AUC) of 0.739 (95% CI 0.684–0.795) and an optimism-corrected AUC of 0.733. It exceeded the blood-derived index model (AUC=0.594, *P* < 0.001) and simplified endoscopic model (AUC = 0.711, *P* = 0.024), but did not differ significantly from the three-item endoscopic model comprising vascular pattern abnormality, bleeding, and erosions/ulcers (AUC = 0.758, *P* = 0.521). At a cutoff of 0.155, Negative predictive value was 0.901. LASSO selected vascular pattern abnormality, bleeding, and mucosal granularity (optimism-corrected AUC = 0.746).

**Discussion:**

The combined model showed moderate discrimination, with modest incremental value from blood-derived indices.

## 1 Introduction

Ulcerative colitis (UC) is a major subtype of inflammatory bowel disease (IBD), characterized by chronic, relapsing inflammation of the intestinal mucosa. The burden of IBD has continued to increase in newly industrialized regions, including Asia (1, 2). An analysis of urban health insurance data reported an IBD incidence of 10.04 per 100,000 person-years in urban China in 2016, indicating that IBD is an increasingly important gastrointestinal disease requiring sustained clinical attention (3). As the number of affected patients grows, improving the early recognition and differential diagnosis of UC in routine care has become an important clinical challenge.

The diagnosis of UC requires integration of clinical presentation, endoscopic and histopathological findings, laboratory results, and follow-up information. Endoscopically, UC commonly presents with continuous mucosal erythema, edema, loss of vascular pattern, erosions, and ulceration. However, infectious enteritis, intestinal tuberculosis, ischemic colitis, and drug-related enteropathy may show similar features. In patients with early disease or atypical endoscopic findings, particularly when histopathological findings are pending or inconclusive, endoscopic appearances alone may be insufficient for an accurate diagnosis (4–6). Cohort studies from China have also reported persistent diagnostic delay in patients with IBD (7).

Fecal calprotectin (FC) is a non-invasive marker of neutrophil-mediated intestinal inflammation and has clinical value in the diagnosis of IBD, assessment of disease activity, and monitoring of relapse. Compared with systemic inflammatory markers such as C-reactive protein (CRP) and erythrocyte sedimentation rate (ESR), FC is more closely associated with local intestinal inflammation and endoscopic activity (8–14). However, FC testing requires appropriate laboratory capacity and stool-sample collection procedures, and its availability varies across healthcare institutions. In real-world practice, its use may also be limited by testing capacity, cost, and sample collection logistics, particularly in resource-constrained settings. Therefore, in retrospective real-world studies, development of UC diagnostic prediction models relying solely on FC or other ideal biomarkers may be impractical. This highlights the need to investigate more accessible and repeatable predictors for clinical risk assessment.

The neutrophil-to-lymphocyte ratio (NLR), platelet-to-lymphocyte ratio (PLR), and systemic immune-inflammation index (SII) can be derived from routine complete blood counts. They are readily available, inexpensive, and reproducible measures that may reflect systemic inflammatory and immune status (15–17). Previous studies have primarily examined their associations with IBD activity, treatment response, and prognosis; evidence regarding their ability to differentiate UC from other intestinal ulcerative diseases remains limited.

For endoscopic assessment, the Ulcerative Colitis Endoscopic Index of Severity (UCEIS) has demonstrated good reliability and value in evaluating endoscopic disease activity (18). However, its standardized use requires appropriate training and complete scoring procedures (19–22). In resource-constrained and real-world settings, more readily recognizable endoscopic features may provide complementary information. Mucosal granularity and mucosal edema are commonly described in endoscopy reports and can be assessed without additional testing or patient burden. Whether routinely reported endoscopic features combined with blood count-derived indices can help distinguish UC from other inflammatory or ulcerative intestinal diseases when FC results or complete standardized endoscopic scores are unavailable in clinical records remains unclear.

Accordingly, we conducted a multicenter, retrospective real-world study using data from four medical centers with two objectives. First, using FC as an index marker, we described the completeness and missingness of inflammation-related variables relevant to the differential diagnosis of UC in routinely collected clinical records, thereby characterizing their recorded availability in real-world care. Second, using predictors that did not require FC results, we assessed the discriminative information obtainable from routine data, including simplified morphological features reported at endoscopy and complete blood count-derived inflammatory indices. We then developed a diagnostic prediction model for UC incorporating mucosal granularity, mucosal edema, NLR, and SII.

Importantly, this model is not intended to replace colonoscopy, histopathology, FC testing, or evaluation for infectious causes. Rather, it is designed to support initial risk assessment and differential diagnostic decision-making using routinely available information when colonoscopy has identified inflammatory or ulcerative lesions but histopathological or FC results are pending or unavailable.

## 2 Materials and methods

### 2.1 Study design and participants

This multicenter retrospective study included patients who underwent an eligible colonoscopy between January 1, 2020, and December 31, 2025, at four participating medical centers: Guilin Hospital of Integrated Traditional Chinese and Western Medicine, the Second Affiliated Hospital of Guangxi University of Chinese Medicine, Minzu Hospital of Guangxi Zhuang Autonomous Region, and Guidong People's Hospital of Guangxi Zhuang Autonomous Region. Data were extracted from the electronic medical records, endoscopy reports, and laboratory information systems of the participating centers.

### 2.2 Eligibility criteria

The inclusion criteria: (1) colonoscopic evidence of congestion, edema, erosions, ulcers, or other inflammatory changes in the colorectal mucosa; (2) an evaluable endoscopy report and essential clinical information; and (3) a documented final clinical diagnosis.

The exclusion criteria: (1) a confirmed colorectal or other intestinal malignancy; (2) inadequate bowel preparation, incomplete colonoscopy, or non-evaluable endoscopic findings; (3) an indeterminate final clinical diagnosis; (4) missing documentation of the final diagnosis or essential endoscopic findings; and (5) duplicate patient records arising from repeated admissions, examinations, or data entry. Repeated records were identified by cross-checking source records after pooling the four-center data; only the first eligible record per patient was retained.

Missing laboratory measurements did not constitute grounds for exclusion from the overall study cohort. The primary comparison of the four prespecified prediction models was conducted in a common complete-case cohort with complete data for all predictors required by the four models.

### 2.3 Outcome ascertainment and predictor definitions

The primary outcome was the final clinical diagnosis of UC recorded in the inpatient or outpatient medical record. During routine care, the diagnosis was established by integrating clinical presentation, colonoscopic findings, histopathological findings and follow-up information when available, and laboratory results. Treating clinicians had access to the endoscopic and laboratory findings, and no independent blinded adjudication of the final diagnosis was performed. Final diagnoses were determined through an integrated assessment of clinical presentation, colonoscopic findings, histopathological findings, laboratory results, and follow-up information. Patients with a final diagnosis of another intestinal inflammatory or ulcerative disease were classified into the non-UC group.

Collected variables included age, sex, disease duration, study center, endoscopic findings, and laboratory measurements. The neutrophil-to-lymphocyte ratio (NLR) was calculated as the absolute neutrophil count divided by the absolute lymphocyte count; the platelet-to-lymphocyte ratio (PLR) as the platelet count divided by the absolute lymphocyte count; and the systemic immune-inflammation index (SII) as the platelet count multiplied by the absolute neutrophil count and divided by the absolute lymphocyte count. Endoscopic severity-related variables, including vascular pattern, bleeding, and erosions/ulcers, were originally recorded as ordinal categories. For the modelling analyses, these variables were dichotomized as absent (score = 0) or present (score > 0). SII was divided by 1,000 before model fitting; therefore, its odds ratio represents the change in the odds of UC per 1,000-unit increase in SII.

Four prespecified prediction models for UC were compared. Model 1 combined mucosal granularity, mucosal edema, NLR, and SII. Model 2 was a simplified endoscopic model comprising mucosal granularity and mucosal edema. Model 3 was a blood-derived inflammatory-index model comprising NLR and SII. Model 4 was a UCEIS-component model comprising dichotomized vascular pattern abnormality, bleeding, and erosions/ulcers; the total UCEIS score was not used.

### 2.4 Statistical analysis

Continuous variables were summarized as medians with interquartile ranges (IQRs), and categorical variables as counts and percentages. Multivariable logistic regression was used to estimate odds ratios (ORs) and 95% confidence intervals (CIs). Pairwise associations among model predictors were assessed using Spearman rank correlation coefficients, and multicollinearity was evaluated using variance inflation factors.

Model discrimination was assessed using the area under the receiver operating characteristic curve (AUC). The 95% CIs for the AUCs were estimated using the DeLong method, and between-model differences were assessed using paired DeLong tests (23).

Overall prediction error was quantified using the Brier score, whereas the Akaike information criterion (AIC) was reported as a relative measure of model fit. An apparent spline-based calibration curve with 95% confidence intervals was plotted for Model 1. The apparent calibration intercept and slope were estimated, and the Hosmer-Lemeshow goodness-of-fit test was performed (24). The optimal probability cutoff was determined by maximising the Youden index. Sensitivity, specificity, positive and negative predictive values, accuracy, and positive and negative likelihood ratios were calculated at this cutoff. To examine the separate contributions of NLR and SII, Model 2 was extended with either index alone in the same complete-case cohort and compared with Model 1 using apparent AUC, Brier score, AIC, and paired DeLong tests.

Decision curve analysis was performed across threshold probabilities ranging from 0.01 to 0.80 (25). Internal validation used 1,000 bootstrap resamples to estimate optimism-corrected AUCs, Brier scores, calibration slopes, and cutoff-based classification measures. For Model 1 model fitting and Youden-index cutoff selection were repeated in each resample (26). Between-center transportability of Model 1 was explored using leave-one-center-out internal-external cross-validation (27). The incremental predictive value of Model 1 over Model 2 was assessed exploratorily using the continuous net reclassification improvement (NRI) and integrated discrimination improvement (IDI), with 95% CIs derived from 2,000 bootstrap resamples (28).

Exploratory LASSO logistic regression was performed using 10-fold cross-validation and the one-standard-error rule. In a sensitivity analysis, multiple imputation by chained equations generated 20 imputed datasets, and LASSO regression was repeated in each dataset (29–31). Variables selected in at least 10 datasets were considered consensus variables. Predicted probabilities were averaged across the 20 fitted models to estimate the apparent AUC and Brier score; bootstrap validation was not performed for this exploratory analysis.

For the four primary models, chained-equation multiple imputation generated 20 datasets under a missing-at-random assumption, using the outcome, center, endoscopic features, demographics, disease duration, and blood counts. Continuous variables were imputed by predictive mean matching, and NLR and SII were recalculated. Each model was refitted in each dataset, and apparent AUCs and Brier scores were averaged across datasets. All analyses were conducted using R version 4.5.1.

### 2.5 Ethics statement

The study was approved by the Ethics Committee of Guilin Hospital of Integrated Traditional Chinese and Western Medicine, which served as the lead ethics committee (approval no. KY-2026-002), and by the ethics committees of Minzu Hospital of Guangxi Zhuang Autonomous Region (approval no. Gui Min Yi Lun Shen Tong Zi [2026] No. 11), Guidong People's Hospital of Guangxi Zhuang Autonomous Region (approval no. GDKY202601), and The Second Affiliated Hospital of Guangxi University of Chinese Medicine (approval no. KY2026-05). The study was conducted in accordance with the Declaration of Helsinki. Because this retrospective study used de-identified clinical records, the requirement for written informed consent was waived by the lead ethics committee.

## 3 Results

### 3.1 Baseline characteristics

A total of 655 patients were included, comprising 135 patients with UC (20.6%) and 520 without UC (79.4%). The non-UC group included infectious enteritis, Crohn's disease, intestinal tuberculosis, ischemic colitis, drug-induced colitis, and other inflammatory or ulcerative intestinal conditions. The cohort included 163 patients (24.9%) from Guilin Hospital of Integrated Traditional Chinese and Western Medicine, 198 (30.2%) from the Second Affiliated Hospital of Guangxi University of Chinese Medicine, 154 (23.5%) from Minzu Hospital of Guangxi Zhuang Autonomous Region, and 140 (21.4%) from Guidong People's Hospital of Guangxi Zhuang Autonomous Region (Table 1).

**Table 1:** Clinical and endoscopic baseline characteristics.

Variable	Overall (*N* = 655)	Non-UC group (*n* = 520)	UC group (*n* = 135)
Clinical characteristics			
Age, years	51.0 (37.0–60.0)	51.0 (38.0–60.0)	50.0 (34.0–61.0)
Male sex	330 (50.4%)	258 (49.6%)	72 (53.3%)
Disease duration, years	0.17 (0.08–0.58); *n* = 627	0.17 (0.08–0.50); *n* = 496	0.25 (0.08–1.00); *n* = 131
Study center, *n* (%)			
Center 1	163 (24.9%)	114 (21.9%)	49 (36.3%)
Center 2	198 (30.2%)	158 (30.4%)	40 (29.6%)
Center 3	154 (23.5%)	124 (23.8%)	30 (22.2%)
Center 4	140 (21.4%)	124 (23.8%)	16 (11.9%)
Endoscopic findings			
Abnormal vascular pattern	293 (44.7%)	188 (36.2%)	105 (77.8%)
Bleeding	64 (9.8%)	34 (6.5%)	30 (22.2%)
Erosions/ulcers	550 (84.0%)	425 (81.7%)	125 (92.6%)
Mucosal granularity	241 (36.8%)	145 (27.9%)	96 (71.1%)
Mucosal edema	450 (68.7%)	338 (65.0%)	112 (83.0%)
Inflammatory polyps	15 (2.3%)	7 (1.3%)	8 (5.9%)
UCEIS-derived score category, *n* (%)			
score 0	36 (5.5%)	30 (5.8%)	6 (4.4%)
score 1–3	581 (88.7%)	477 (91.7%)	104 (77.0%)
score 4–6	37 (5.6%)	13 (2.5%)	24 (17.8%)
score 7–8	1 (0.2%)	0 (0.0%)	1 (0.7%)

Data are presented as median (IQR) or *n* (%). For variables with missing data, the available sample size is shown in the corresponding cell. Vascular pattern, bleeding, and erosions/ulcers were dichotomized as absent (score = 0) or present (score > 0). IQR, interquartile range; UC, ulcerative colitis; UCEIS, Ulcerative Colitis Endoscopic Index of Severity.

Descriptive comparisons of baseline characteristics showed that vascular pattern abnormality, bleeding, erosions/ulcers, mucosal granularity, and mucosal edema were more frequent in the UC group than in the non-UC group. Median platelet count, NLR, PLR, and SII values were also numerically higher in the UC group (Tables 1, 2). The distributions of NLR, PLR, and SII are shown in Figure 1.

**Table 2:** Baseline laboratory characteristics.

Laboratory index	Overall (*N* = 655)	Non-UC (*n* = 520)	UC (*n* = 135)
Hemoglobin, g/L	129.0 (113.0–140.0); *n* = 459	130.0 (115.0–142.0); *n* = 354	125.0 (106.0–137.0); *n* = 105
Neutrophil count, x10^9^/L	4.22 (3.20–5.30); *n* = 457	4.12 (3.18–5.13); *n* = 354	4.54 (3.30–6.40); *n* = 103
Lymphocyte count, x10^9^/L	1.68 (1.30–2.15); *n* = 457	1.69 (1.24–2.15); *n* = 354	1.62 (1.38–2.14); *n* = 103
Mean platelet volume, fL	9.60 (8.89–10.31); *n* = 456	9.60 (8.90–10.30); *n* = 353	9.60 (8.70–10.46); *n* = 103
Platelet count, x10^9^/L	249.0 (203.5–312.0); *n* = 459	245.5 (200.2–304.5); *n* = 354	270.0 (214.0–341.0); *n* = 105
Albumin, g/L	40.6 (37.7–44.2); *n* = 447	40.8 (37.8–44.5); *n* = 343	39.8 (36.2–42.8); *n* = 104
NLR	2.46 (1.76–3.58); *n* = 457	2.37 (1.74–3.46); *n* = 354	2.87 (1.97–4.19); *n* = 103
PLR	148.56 (110.98–210.11); *n* = 455	145.87 (106.19–203.08); *n* = 352	163.38 (125.84–231.04); *n* = 103
SII, x10^9^/L	620.30 (400.33–942.68); *n* = 455	596.20 (386.18–897.77); *n* = 352	692.86 (512.29–1194.72); *n* = 103
CRP/albumin ratio	0.13 (0.07–0.60); *n* = 252	0.13 (0.06–0.45); *n* = 188	0.17 (0.12–0.67); *n* = 64
Fecal calprotectin, ug/g	384.0 (237.4–523.5); *n* = 6	30.0; *n* = 1	445.0 (323.0–549.7); *n* = 5
ESR, mm/h	23.0 (12.0–43.0); *n* = 161	21.0 (10.5–39.2); *n* = 104	31.0 (15.0–47.0); *n* = 57
CRP, mg/L	5.00 (2.90–24.50); *n* = 255	5.00 (2.65–19.90); *n* = 191	6.12 (5.00–27.02); *n* = 64

Data are presented as median (IQR). For variables with missing data, the available sample size is shown in the corresponding cell. CRP, C-reactive protein; ESR, erythrocyte sedimentation rate; IQR, interquartile range; NLR, neutrophil-to-lymphocyte ratio; PLR, platelet-to-lymphocyte ratio; SII, systemic immune-inflammation index; UC, ulcerative colitis.

**Figure 1:**
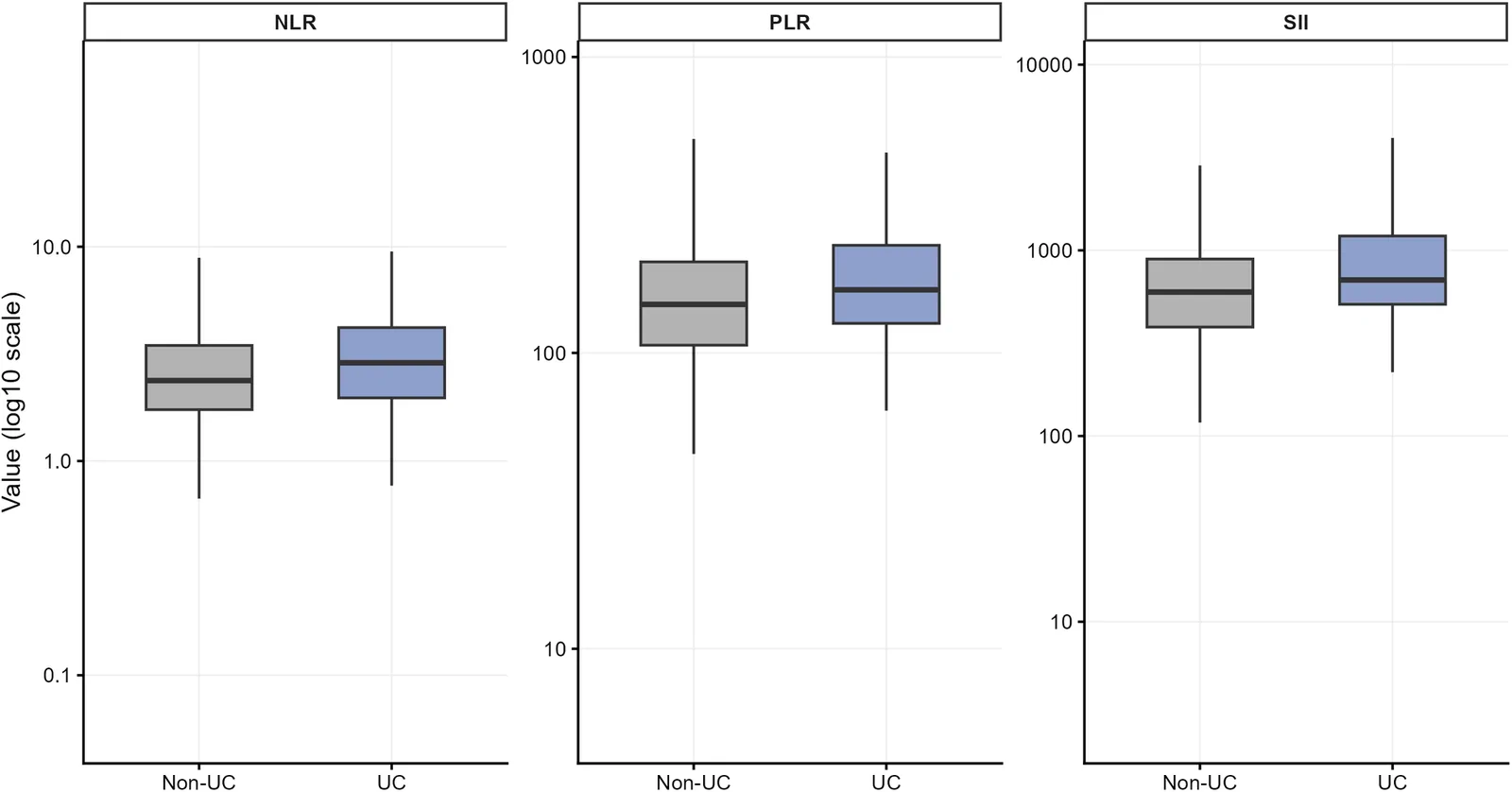
Distribution of blood-based inflammatory indices in the UC and non-UC groups. NLR, neutrophil-to-lymphocyte ratio; PLR, platelet-to-lymphocyte ratio; SII, systemic immune-inflammation index; UC, ulcerative colitis.

Assessment of missing data among the candidate variables showed substantial missingness for FC, ESR, CRP, and the CRP-to-albumin ratio. FC results were documented for only 6 of 655 patients (0.9%); therefore, FC was excluded from the primary modelling analysis. The distribution of missing data across the candidate variables is shown in Figure 2.

**Figure 2:**
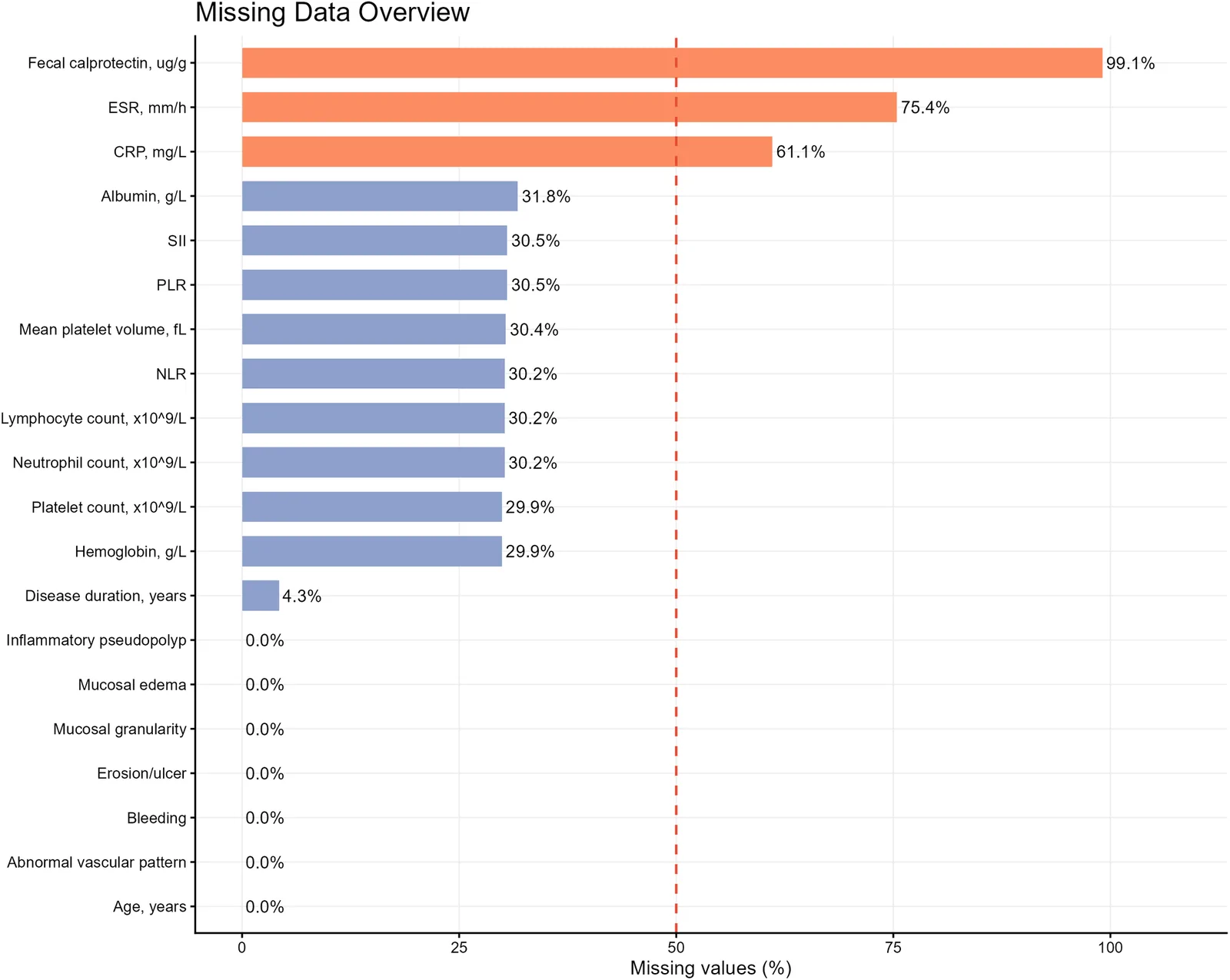
Missingness among candidate variables. CRP, C-reactive protein; ESR, erythrocyte sedimentation rate; NLR, neutrophil-to-lymphocyte ratio; PLR, platelet-to-lymphocyte ratio; SII, systemic immune-inflammation index.

### 3.2 Predictive performance of the prespecified models

The common complete-case cohort comprised 455 patients, of whom 103 had UC, corresponding to a prevalence of 22.6%.The remaining 200 patients had missing blood-count data required to calculate NLR or SII and were therefore omitted from the common complete-case analysis. ROC analysis showed that Model 4 (the UCEIS-component model) had the numerically highest apparent AUC of 0.758 (95% CI 0.705–0.811). The apparent AUCs were 0.739 (95% CI 0.684–0.795) for Model 1, 0.711 (95% CI 0.661–0.762) for Model 2, and 0.594 (95% CI 0.531–0.657) for Model 3 (Table 3; Figure 3).

**Table 3:** Apparent and bootstrap-corrected performance of the four prespecified models.

Model	N/UC cases	Apparent AUC	AUC 95% CI	Apparent Brier score	Optimism-corrected AUC	Optimism-corrected Brier score	AIC	MI mean apparent AUC	MI mean apparent Brier score
Model 1: Mucosal granularity+mucosal edema+SII+NLR	455/103	0.739	0.684–0.795	0.151	0.733	0.155	436.779	0.750	0.139
Model 2: Mucosal granularity+mucosal edema	455/103	0.711	0.661–0.762	0.156	0.709	0.158	440.592	0.730	0.142
Model 3: NLR+SII	455/103	0.594	0.531–0.657	0.172	0.584	0.175	485.347	0.578	0.162
Model 4: UCEIS-component model	455/103	0.758	0.705–0.811	0.142	0.754	0.145	421.882	0.758	0.132

All models were evaluated in the common complete-case cohort of 455 patients, including 103 patients with UC. The multiple-imputation sensitivity analysis included all 655 patients, including 135 patients with UC; the reported MI estimates represent mean imputation-specific apparent AUCs and Brier scores across 20 imputed datasets. Model 4 comprised dichotomized vascular pattern abnormality, bleeding, and erosions/ulcers. Optimism correction was based on 1,000 bootstrap resamples and applied only to the complete-case models. AIC values also refer to the complete-case models. Lower Brier scores and AIC values indicate better performance. AIC, Akaike information criterion; AUC, area under the receiver operating characteristic curve; CI, confidence interval;MI, multiple imputation; NLR, neutrophil-to-lymphocyte ratio; SII, systemic immune-inflammation index; UC, ulcerative colitis; UCEIS, Ulcerative Colitis Endoscopic Index of Severity.

**Figure 3:**
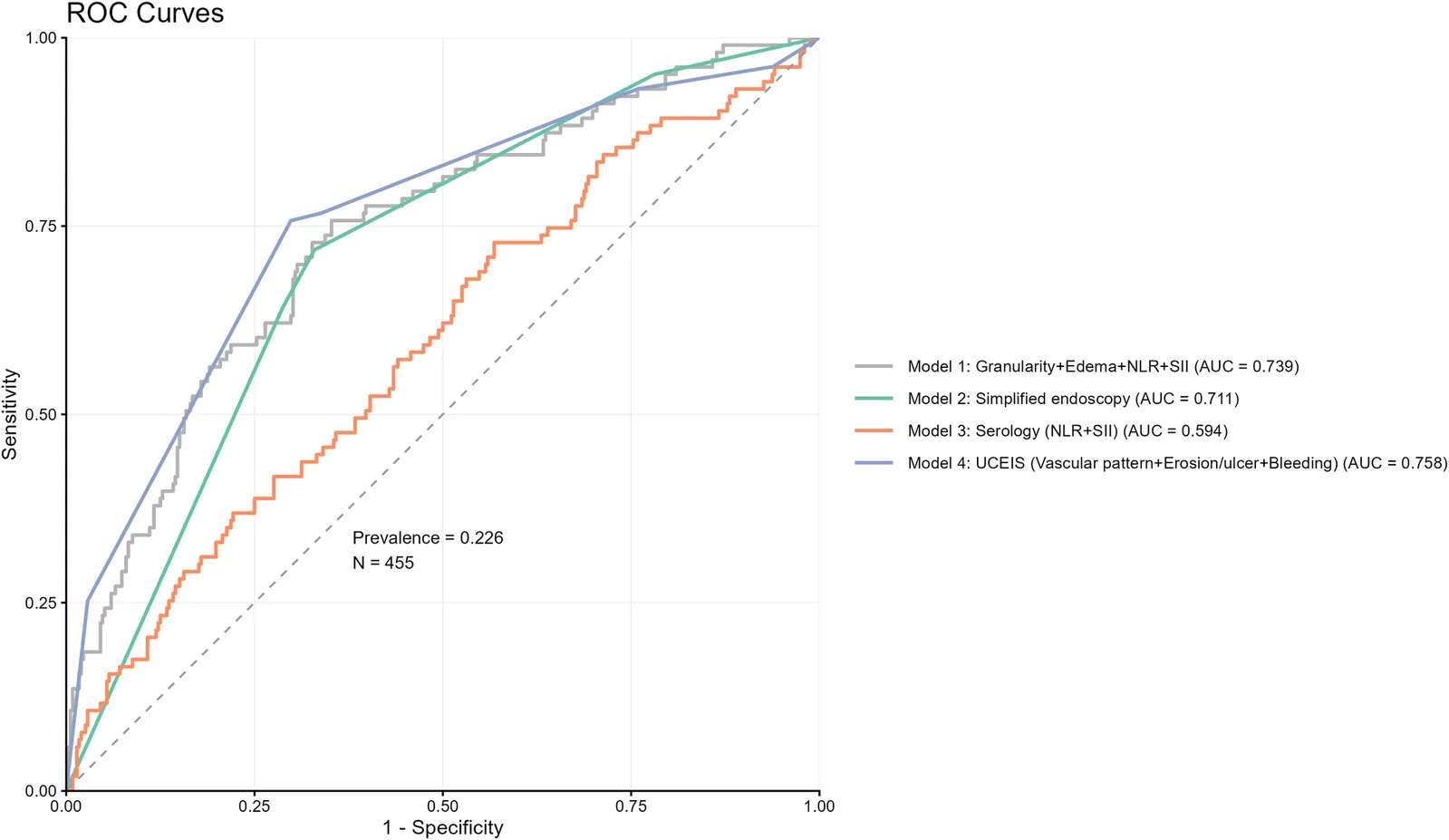
Receiver operating characteristic curves for the four prespecified models. Model 1 included mucosal granularity, mucosal edema, NLR, and SII; Model 2 included mucosal granularity and mucosal edema; Model 3 included NLR and SII; and Model 4 included the dichotomized UCEIS components of vascular pattern abnormality, bleeding, and erosions/ulcers. AUC, area under the receiver operating characteristic curve; NLR, neutrophil-to-lymphocyte ratio; SII, systemic immune-inflammation index; UCEIS, Ulcerative Colitis Endoscopic Index of Severity.

Paired DeLong tests showed that the AUC of Model 1 was higher than those of Model 2 (difference in AUC = 0.028, *P* = 0.024) and Model 3 (difference in AUC = 0.145, *P* < 0.001). The AUCs of Models 1 and 4 did not differ significantly (difference in AUC = −0.019, *P* = 0.521) (Table 4).

**Table 4:** Paired DeLong comparisons of model AUCs.

Comparison	Model 1 AUC	Comparator AUC	Difference in AUC	Standard error	Z value	*P* value
Model 1 vs. Model 2	0.739	0.711	0.028	0.012	2.253	0.024
Model 1 vs. Model 3	0.739	0.594	0.145	0.033	4.427	<0.001
Model 1 vs. Model 4	0.739	0.758	−0.019	0.029	−0.641	0.521

Differences in AUC were calculated as the AUC of Model 1 minus that of the comparator model. *P* values were obtained using paired DeLong tests. AUC, area under the receiver operating characteristic curve; SE, standard error.

In a sensitivity analysis using the same 455 patients, the apparent AUC was 0.692 for Model 2 plus NLR and 0.731 for Model 2 plus SII, compared with 0.739 for Model 1. NLR and SII were correlated (Spearman *ρ*=0.862; VIFs 3.25 and 3.26, respectively). Compared with Model 2 plus SII, Model 1 had a lower Brier score (0.151 vs. 0.154) and AIC (436.78 vs. 440.09), but its AUC was not significantly higher (paired DeLong *P* = 0.480).

In an exploratory incremental-value analysis, Model 2 was used as the reference model to assess the additional predictive information provided by NLR and SII. Compared with Model 2, Model 1 yielded a continuous net reclassification improvement of 0.240 (bootstrap 95% CI 0.044–0.458) and an integrated discrimination improvement of 0.021 (bootstrap 95% CI 0.006–0.038). These exploratory results suggested an apparent improvement when NLR and SII were added together to Model 2, but did not establish a separate incremental contribution from each index.

Within the development cohort, maximisation of the Youden index identified an apparent optimal predicted-probability cutoff of 0.155 for Model 1. At this cutoff, sensitivity was 0.757, specificity was 0.648, positive predictive value was 0.386, negative predictive value was 0.901, accuracy was 0.673, and the Youden index was 0.405.

Model 1 had an apparent Brier score of 0.151, an apparent calibration intercept of approximately 0, and an apparent calibration slope of 1.000. Bootstrap correction for optimism yielded an AUC of 0.733, a Brier score of 0.155, and a calibration slope of 0.952. After bootstrap optimism correction, the corresponding sensitivity, specificity, PPV, and NPV for the model-and-cutoff selection procedure were 0.734, 0.640, 0.372, and 0.893, respectively (Table 5).

**Table 5:** Performance, calibration, and cutoff-based classification measures for model 1.

Category	Measure	Estimate
Sample and model	Analysis cohort	*N* = 455; 103 UC cases (22.6%)
Discrimination	AUC	0.739 (95% CI 0.684–0.795)
Apparent calibration	Brier score	0.151
Apparent calibration	Calibration intercept	−0.000
Apparent calibration	Calibration slope	1.000
Internal validation	Optimism-corrected AUC	0.733
Internal validation	Optimism-corrected Brier score	0.155
Internal validation	Optimism-corrected calibration slope	0.952
Classification threshold	Optimal cutoff	0.155
Performance at the cutoff	Sensitivity	0.757 (95% CI 0.666–0.830)
Performance at the cutoff	Specificity	0.648 (95% CI 0.596–0.696)
Performance at the cutoff	PPV	0.386 (95% CI 0.322–0.455)
Performance at the cutoff	NPV	0.901 (95% CI 0.858–0.932)
Performance at the cutoff	Accuracy	0.673 (95% CI 0.628–0.714)
Performance at the cutoff	Youden index	0.405
Performance at the cutoff	LR+	2.150 (95% CI 1.797–2.571)
Performance at the cutoff	LR-	0.375 (95% CI 0.264–0.532)
Apparent calibration	Hosmer-Lemeshow test	*χ*^2^ = 5.148; df = 8; *P* = 0.742
Bootstrap-corrected classification	Optimism-corrected sensitivity	0.734
Bootstrap-corrected classification	Optimism-corrected specificity	0.640
Bootstrap-corrected classification	Optimism-corrected PPV	0.372
Bootstrap-corrected classification	Optimism-corrected NPV	0.893

Apparent cutoff-based estimates were derived from apparent predictions in the common complete-case development cohort [*N* = 455; 103 UC cases (22.6%)] and were not corrected for optimism. The four optimism-corrected cutoff-based measures were estimated using 1,000 bootstrap resamples, with model fitting and cutoff selection repeated in each resample. The apparent optimal cutoff was selected by maximizing the Youden index. The optimism-corrected calibration slope was estimated using 1,000 bootstrap resamples. Predictive values are dependent on the UC prevalence in the study cohort. CI, confidence interval; LR+, positive likelihood ratio; LR−, negative likelihood ratio; NPV, negative predictive value; PPV, positive predictive value; UC, ulcerative colitis.

### 3.3 Decision curve analysis and calibration

Apparent decision curve analysis showed that Models 1 and 4 provided positive net benefit across threshold probabilities of approximately 0.10–0.30 and generally provided greater net benefit than the treat-all and treat-none strategies. Model 3 provided comparatively lower net benefit (Figure 4).

**Figure 4:**
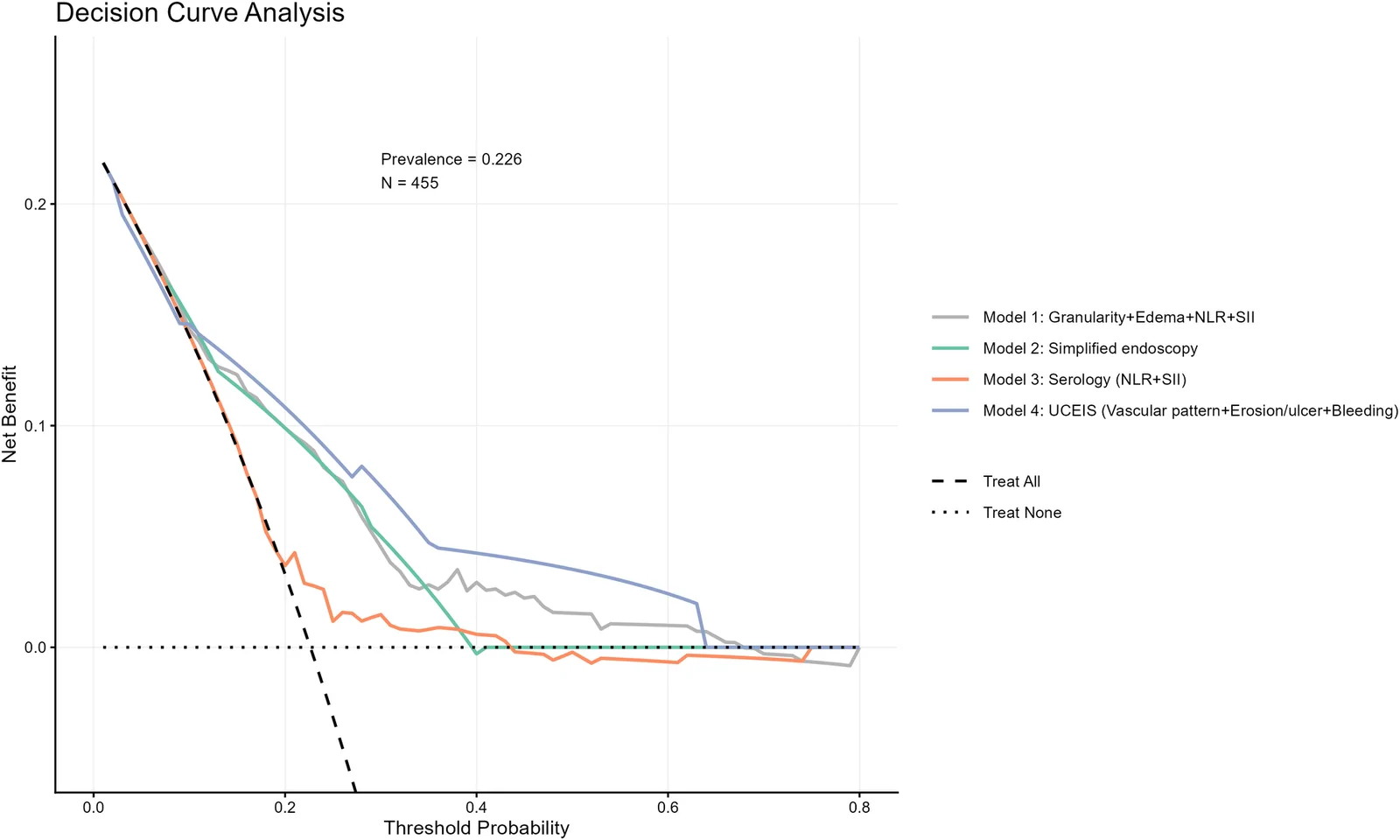
Decision curve analysis for the four prespecified models. Apparent decision curve analysis of the four prespecified diagnostic prediction models for ulcerative colitis in the common complete-case cohort. Net benefit was evaluated across threshold probabilities from 0.01 to 0.80. Model 1 included mucosal granularity, mucosal edema, NLR, and SII; Model 2 included mucosal granularity and mucosal edema; Model 3 included NLR and SII; and Model 4 included the dichotomized UCEIS components. The dashed and dotted black lines represent the treat-all and treat-none strategies, respectively. At a given threshold probability, a higher curve indicates greater estimated net benefit. NLR, neutrophil-to-lymphocyte ratio; SII, systemic immune-inflammation index; UC, ulcerative colitis; UCEIS, Ulcerative Colitis Endoscopic Index of Severity.

The flexible apparent calibration curve for Model 1 was broadly close to the ideal line, although the 95% confidence intervals widened at higher predicted probabilities (Figure 5). The Hosmer-Lemeshow test provided no evidence of lack of fit (*χ*^2^ = 5.148, df = 8, *P* = 0.742).

**Figure 5:**
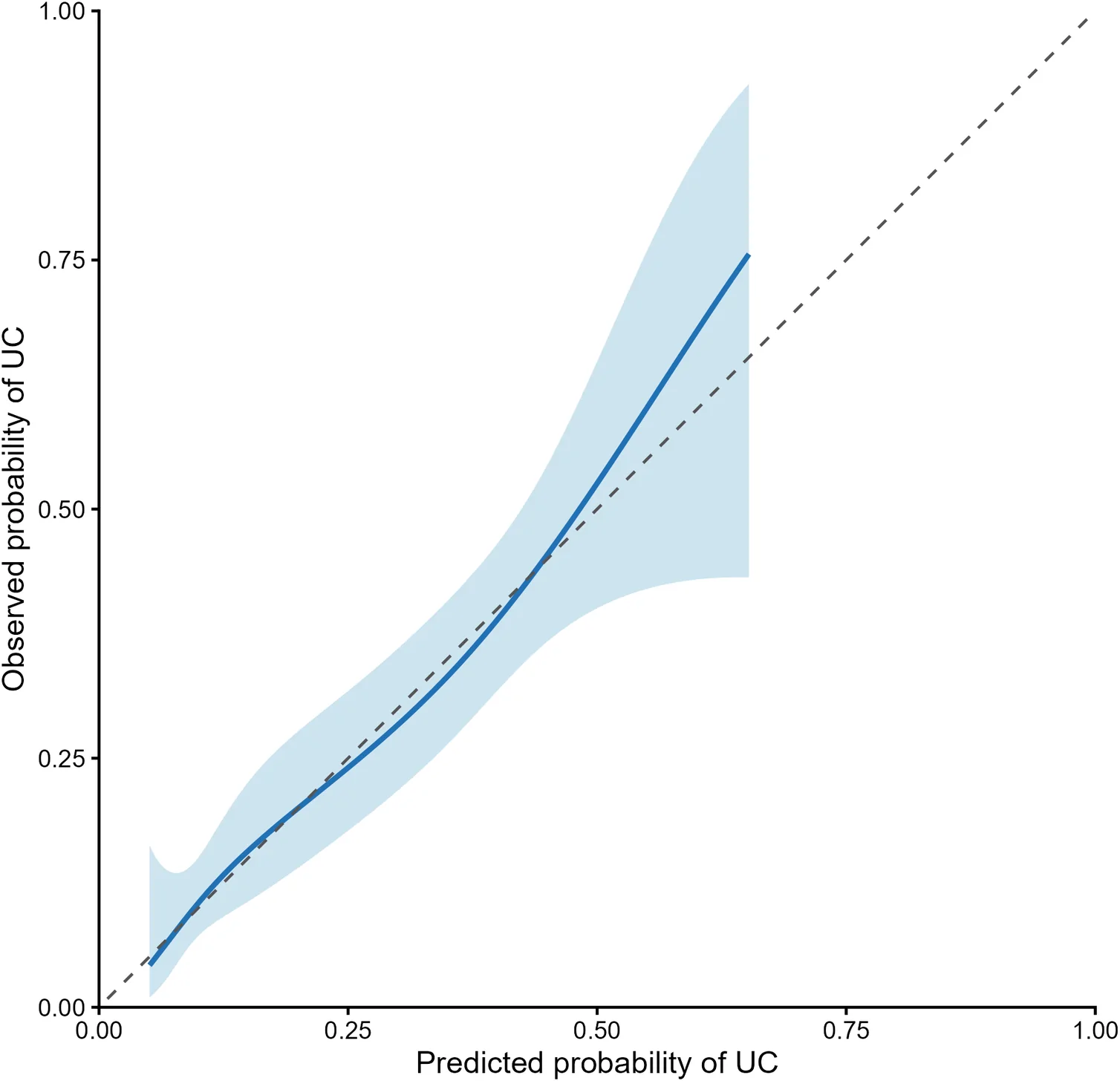
Apparent calibration curve for model 1. The solid line shows the spline estimate, the shaded band its pointwise 95% confidence interval, and the dashed line ideal calibration. The curve spans the 1st–99th percentiles of predicted probabilities.

### 3.4 Multivariable logistic regression analysis

A multivariable logistic regression model was fitted using the four predictors prespecified for Model 1. In the multivariable model, mucosal granularity was associated with higher odds of a final UC diagnosis (adjusted OR = 4.817, 95% CI 2.928–7.924, *P* < 0.001). Each 1,000-unit increase in SII was also associated with higher odds of UC (adjusted OR = 1.774, 95% CI 1.139–2.762, *P* = 0.011). Neither mucosal edema (adjusted OR = 1.813, 95% CI 0.914–3.597, *P* = 0.089) nor NLR (adjusted OR = 0.877, 95% CI 0.751–1.023, *P* = 0.095) reached statistical significance (Figure 6). For Model 1, let G and E denote mucosal granularity and mucosal edema, respectively (1 = present, 0 = absent). The fitted linear predictor was *η* = −2.634314 + 1.572137G + 0.595037E - 0.131572NLR + 0.573037(SII/1000), and the predicted probability of UC was 1/[1 + exp(-*η*)].

**Figure 6:**
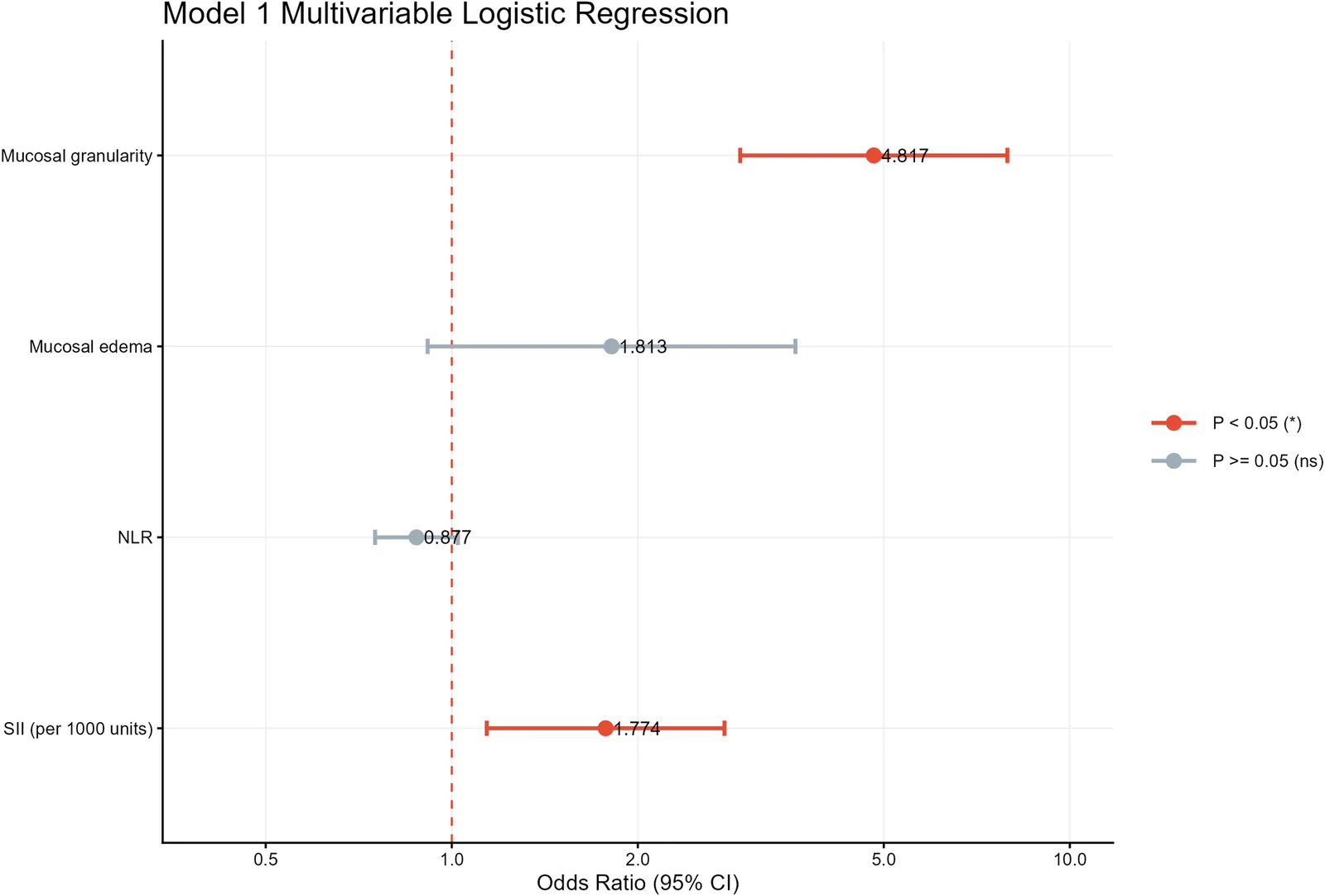
Multivariable logistic regression results for model 1. Multivariable logistic regression estimates for Model 1 in the common complete-case cohort. CI, confidence interval; NLR, neutrophil-to-lymphocyte ratio; OR, odds ratio; SII, systemic immune-inflammation index.

Although SII and NLR were strongly correlated (Figure 7), their variance inflation factors were 3.26 and 3.25, respectively. The variance inflation factors for mucosal granularity and mucosal edema were close to 1, providing no evidence of severe multicollinearity. A static nomogram based on Model 1 was constructed to illustrate how the four predictors contributed to the estimated individual probability of UC (Figure 8).

**Figure 7:**
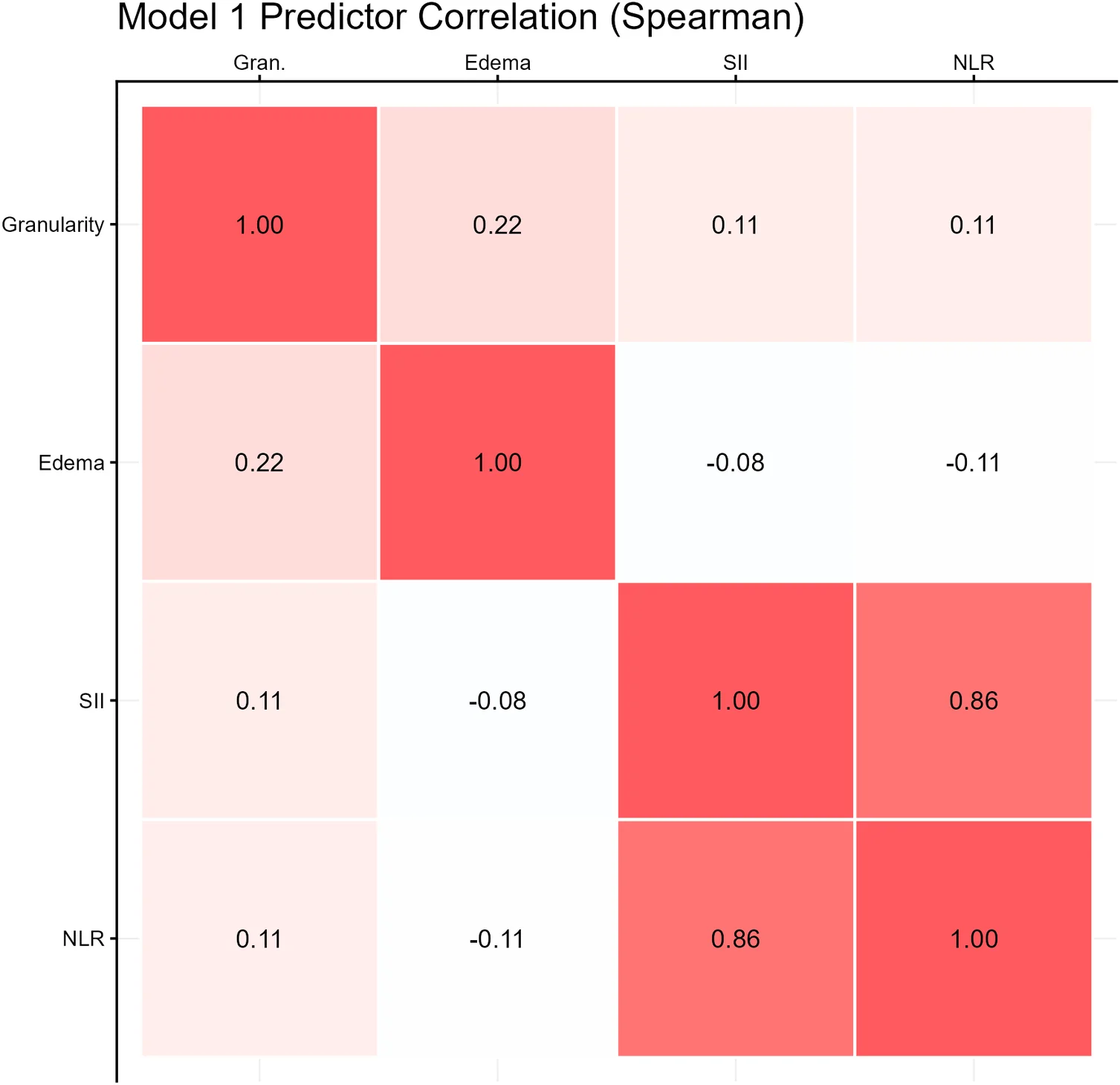
Spearman correlation heatmap for the predictors included in model 1. NLR, neutrophil-to-lymphocyte ratio; SII, systemic immune-inflammation index.

**Figure 8:**
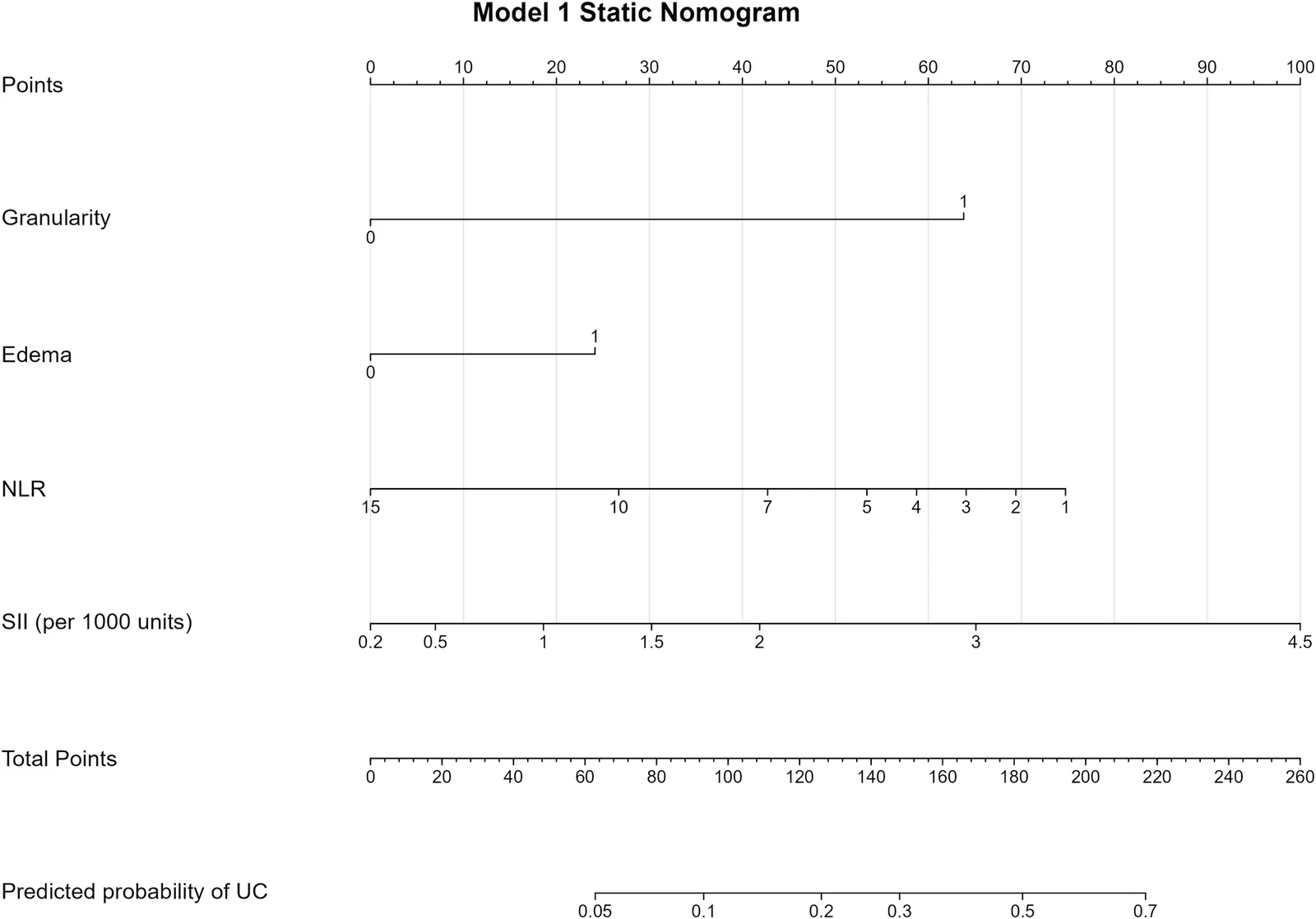
Static nomogram derived from model 1. Nomogram derived from Model 1 for estimating the probability of a final diagnosis of ulcerative colitis. Mucosal granularity and mucosal edema were coded as absent (0) or present (1); SII was scaled per 1,000-unit increase. NLR, neutrophil-to-lymphocyte ratio; SII, systemic immune-inflammation index; UC, ulcerative colitis.

### 3.5 Internal validation and between-center transportability

Bootstrap internal validation using 1,000 resamples showed only small decreases from the apparent AUCs. The optimism-corrected AUCs were 0.733 for Model 1, 0.709 for Model 2, 0.584 for Model 3, and 0.754 for Model 4(Figure 9).

**Figure 9:**
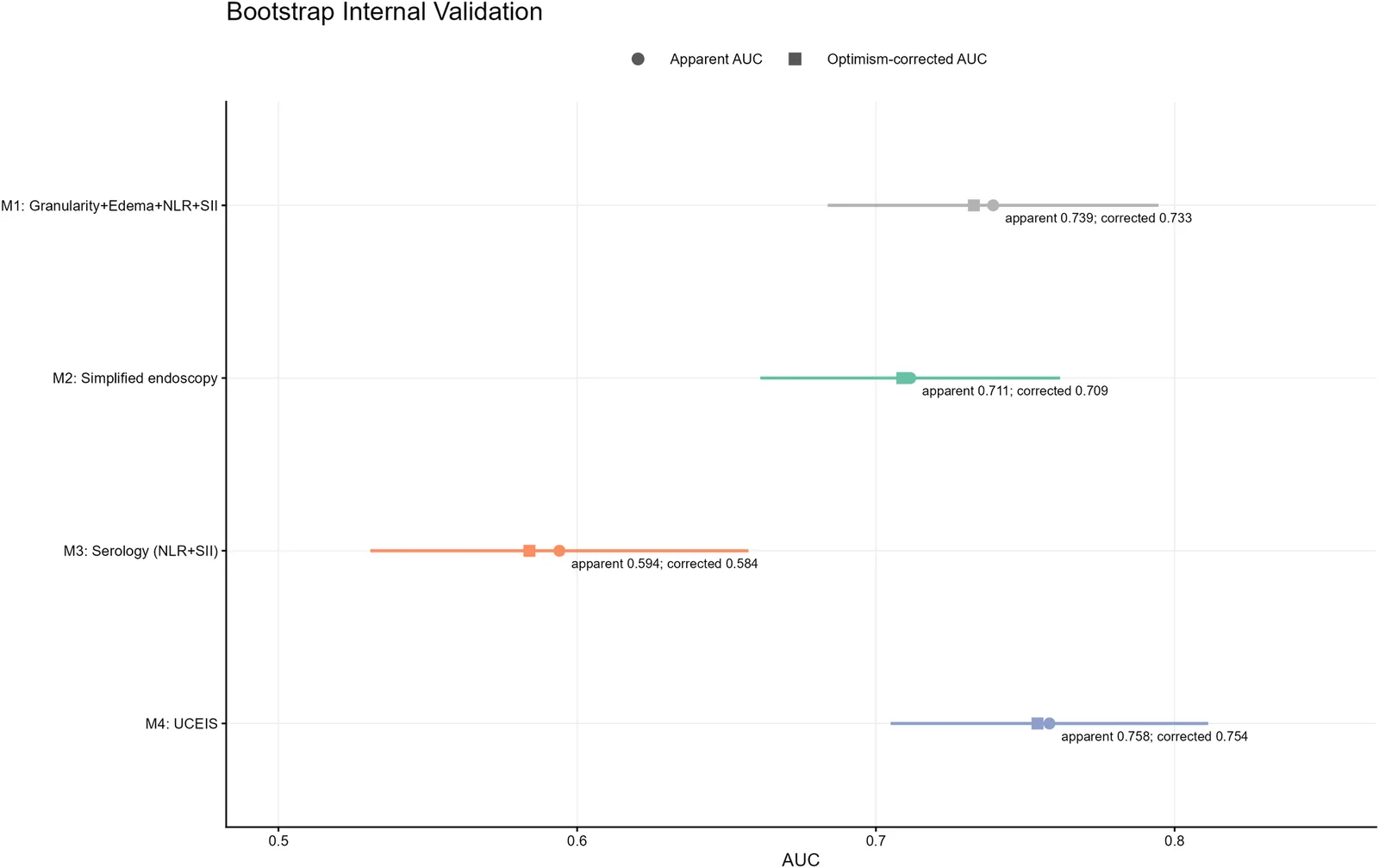
Bootstrap internal validation of the four prespecified models. Apparent and optimism-corrected areas under the receiver operating characteristic curve for the four prespecified models. Model 1 included mucosal granularity, mucosal edema, NLR, and SII; Model 2 included mucosal granularity and mucosal edema; Model 3 included NLR and SII; and Model 4 included the dichotomized UCEIS components. AUC, area under the receiver operating characteristic curve; NLR, neutrophil-to-lymphocyte ratio; SII, systemic immune-inflammation index; UCEIS, Ulcerative Colitis Endoscopic Index of Severity. Horizontal lines indicate 95% confidence intervals for the apparent AUCs.

In leave-one-center-out internal-external cross-validation, the AUC of Model 1 ranged from 0.673 to 0.772 across the four held-out centers, indicating some between-center variation in discrimination (Figure 10). The held-out validation set from Guidong People's Hospital of Guangxi Zhuang Autonomous Region included only 54 complete cases, seven of whom had UC; therefore, its hospital-specific AUC estimate should be interpreted cautiously.

**Figure 10:**
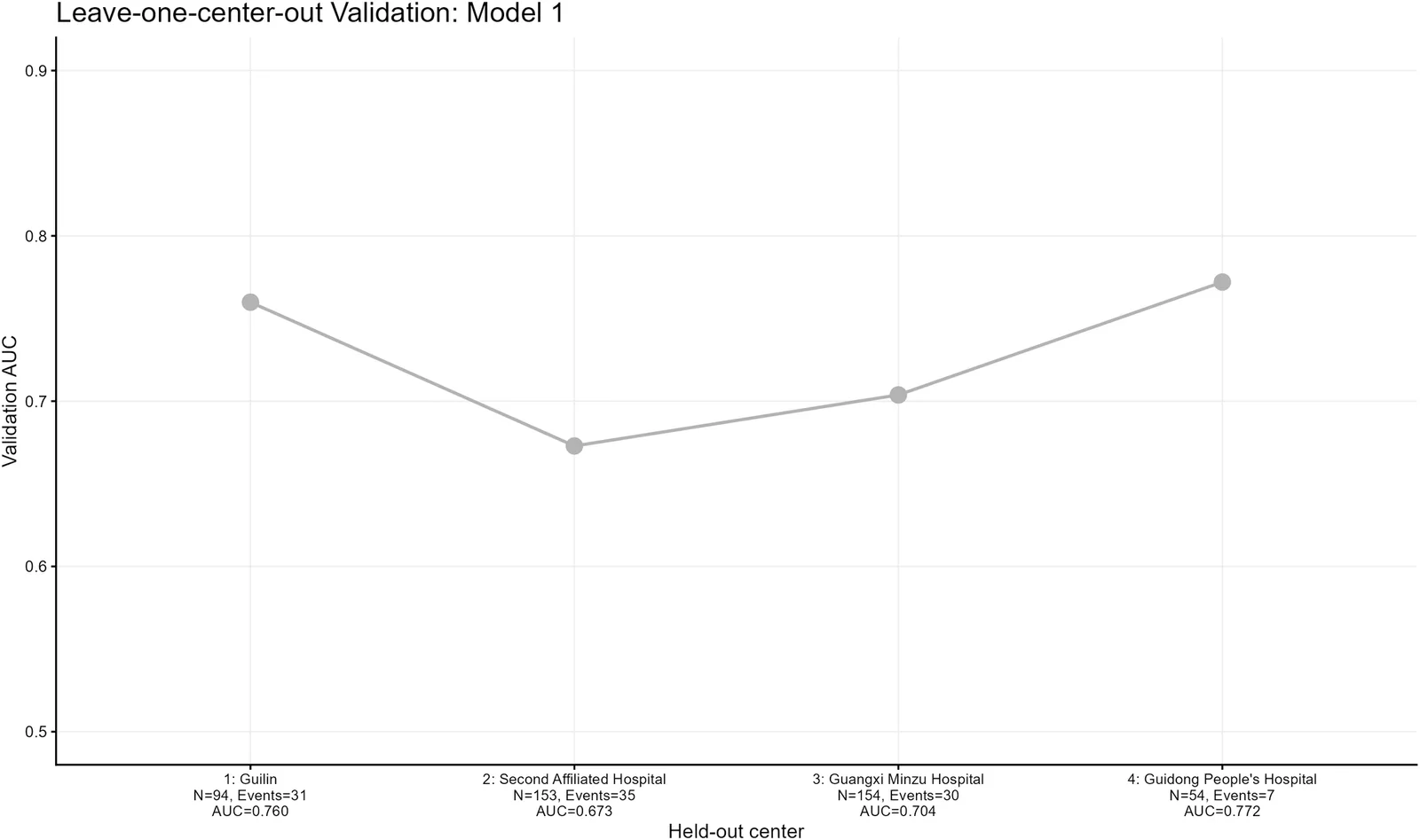
Leave-one-center-out internal-external cross-validation of Model 1. Model 1 was refitted using data from three centers and evaluated in the remaining center, repeated across all four centers. Points show the held-out AUCs. N, sample size; Events, number of UC cases; AUC, area under the receiver operating characteristic curve; UC, ulcerative colitis.

### 3.6 Exploratory variable selection and sensitivity analysis

Exploratory variable selection using LASSO logistic regression was performed in the complete-case cohort for the candidate predictors (*N* = 429; 101 UC cases). Based on 10-fold cross-validation and the one-standard-error rule, the LASSO model selected three predictors: vascular pattern abnormality, bleeding, and mucosal granularity (Supplementary Figures S1 and S2).

These predictors were subsequently entered into an unpenalised multivariable logistic regression model. All three were associated with a final diagnosis of UC: the adjusted OR was 2.623 for vascular pattern abnormality (95% CI 1.490–4.618), 3.318 for bleeding (95% CI 1.702–6.471), and 3.404 for mucosal granularity (95% CI 2.014–5.754; *P* < 0.001 for all) (Supplementary Figure S3). The complete-case LASSO-derived model had an apparent AUC of 0.751, a Brier score of 0.148, and an optimism-corrected AUC of 0.746.

In the exploratory multiple-imputation analysis involving LASSO variable selection, the full cohort of 655 patients, including 135 with UC, was analyzed. The consensus variables were vascular pattern abnormality, mucosal granularity, platelet count, and bleeding. Averaging the individual predicted probabilities across the 20 imputed-dataset models yielded an apparent AUC of 0.805 and a Brier score of 0.127 (Supplementary Figure S4). These exploratory findings showed that vascular pattern abnormality, bleeding, and mucosal granularity were selected under both the complete-case and multiple-imputation approaches. In the separate multiple-imputation sensitivity analysis of the four primary comparison models using the full cohort, the mean imputation-specific apparent AUCs were 0.750, 0.730, 0.578, and 0.758 for Models 1–4, respectively; the corresponding mean Brier scores were 0.139, 0.142, 0.162, and 0.132.

## 4 Discussion

This study first assessed the completeness of inflammatory-marker data in routine clinical records and then evaluated whether routinely reported endoscopic features combined with peripheral blood inflammatory indices could support estimation of the probability of UC. FC was documented in only 6 of 655 patients (0.9%). Mucosal granularity was associated with a final diagnosis of UC in the prespecified multivariable logistic regression model and was also retained in the exploratory LASSO analysis. Model 1, which incorporated mucosal granularity, mucosal edema, NLR, and SII, showed moderate discrimination. Its AUC was numerically lower than that of the three-item UCEIS-component model, although the difference was not statistically significant.

In the common complete-case cohort, Model 1 had an apparent AUC of 0.739 and an optimism-corrected AUC of 0.733. Compared with the simplified endoscopic model comprising mucosal granularity and mucosal edema, adding NLR and SII increased the AUC by 0.028, with a statistically significant difference in the paired DeLong test (*P* = 0.024). Nevertheless, the absolute improvement was modest and was estimated in the same development cohort; therefore, the incremental predictive value of these peripheral blood indices requires confirmation in an independent population. At the apparent optimal cutoff, Model 1 had a negative predictive value of 0.901 and a positive predictive value of 0.386. This profile suggests that the model may support preliminary risk stratification among patients with inflammatory or ulcerative lesions detected at colonoscopy. However, because predictive values depend on disease prevalence and the cutoff was derived from the development data, the model should not be used alone either to confirm or to exclude UC.

### 4.1 Availability of inflammatory biomarker results

FC is an important non-invasive biomarker in the management of IBD. The ACG guideline, the AGA guideline on biomarkers, and the Chinese consensus on the diagnosis and management of IBD all recognise its role in assessing disease activity, monitoring treatment response, and estimating the risk of relapse (4, 8, 32). A systematic review and meta-analysis by Mosli et al (10). found that fecal biomarkers, including FC and fecal lactoferrin, generally performed better than blood-based inflammatory markers for detecting endoscopic activity in IBD. Similarly, Schoepfer et al. reported that FC correlated more closely with endoscopic disease activity in UC than CRP, white blood cell count, or platelet count (12).

The main contribution of this study is to characterize the availability and missingness of FC results in this real-world cohort, rather than to re-establish the clinical value of FC.Among 655 patients with inflammatory or ulcerative intestinal lesions across the four participating centers, only six had a recorded FC result, corresponding to a documentation rate of 0.9%. CRP and ESR data were also missing for 61.1% and 75.4% of patients, respectively. Because FC results were for only six patients, FC could not be included in the primary modelling analysis. FC testing was performed through an external laboratory service. Missing results could reflect tests not being ordered or performed, or results not being documented in the available records. These findings highlight a substantial gap between guideline recommendations regarding inflammatory biomarker assessment and routine clinical documentation at the participating regional centers (33, 34). However, they should not be interpreted as an estimate of national FC testing coverage. Rather, they suggest that limited availability of FC and other recommended biomarkers may constrain both retrospective model development and timely risk assessment in some real-world clinical settings.

Against this background, the present study did not seek to replace FC with routinely available markers. Rather, it evaluated the extent to which routinely documented endoscopic features and complete blood count-derived inflammatory indices could support UC diagnostic assessment when FC, CRP, or ESR results were not available for analysis. A common complete-case cohort was used to ensure that the four prespecified models were evaluated in the same patients, thereby avoiding differences in model performance attributable solely to differences in the analyzed populations. Nevertheless, complete-case analysis may introduce selection bias if patients with complete data differ systematically from those with missing data. Because multiple imputation depends on assumptions regarding the missing-data mechanism and the specification of the imputation model, it was treated as an exploratory sensitivity analysis and was not used to replace the primary findings from the complete-case analysis (29–31).

### 4.2 Adjunctive value of mucosal granularity and simplified endoscopic features

Colonoscopy and histopathological assessment remain central to the diagnosis and differential diagnosis of UC. The UCEIS is a standardized instrument for grading endoscopic disease severity and has demonstrated good reliability and validity in assessing disease activity (18, 19). Previous systematic reviews have indicated that endoscopic scoring systems support the standardized assessment of disease activity in UC, although they vary in complexity, interobserver reliability, and suitability for different clinical settings. More recently, Bergqvist et al. proposed a simplified endoscopic score for UC, highlighting the continuing need for assessment tools that are more practical and reproducible in routine endoscopic practice (21).

Mucosal granularity is not assessed as a separate component in either the UCEIS or the Mayo Endoscopic Score, the two most widely used endoscopic scoring systems for UC.Nevertheless, it is frequently documented in routine endoscopy reports, can be identified directly during colonoscopy, and requires no additional testing. In the present study, mucosal granularity was associated with a final diagnosis of UC after adjustment for the other predictors in Model 1 (OR = 4.817, *P* < 0.001) and was also selected in the exploratory LASSO analysis. These findings suggest that, among patients with inflammatory or ulcerative lesions detected at colonoscopy, mucosal granularity may provide useful information for UC risk assessment, particularly when FC results or complete standardized endoscopic scores are unavailable.

These findings should not be interpreted as evidence that mucosal granularity can replace the UCEIS or histopathological assessment. Rather, it should be regarded as a supportive endoscopic clue that may contribute to initial UC risk assessment. Because of the retrospective design, information on mucosal granularity was derived primarily from routine endoscopy reports, without centralized image review or formal assessment of interobserver agreement. Its documentation may therefore have been influenced by subjective interpretation and center-specific reporting practices. Prospective studies incorporating standardized image acquisition, independent blinded assessment by multiple endoscopists, and interobserver agreement analysis using kappa statistics are needed to refine its definition and determine its reproducibility and practical value in differentiating UC from non-UC conditions (35, 36).

### 4.3 Diagnostic value and limitations of peripheral blood-derived inflammatory indices

NLR, PLR, and SII can all be calculated from routine complete blood count data and are inexpensive, widely available, and suitable for repeated measurement. Their potential role in the assessment of IBD has received increasing attention. Yan et al. (15) reported that SII may serve as a marker of disease activity in UC and was associated with clinical, endoscopic, and histological inflammatory activity. Feng et al. (16) found that NLR and PLR were elevated in patients with active UC and showed some ability to distinguish active disease from remission. A systematic review similarly reported associations between peripheral blood-derived indices, including NLR and PLR, and inflammatory activity in IBD (17). However, their discriminatory performance may vary according to the study population, disease activity, comparator-group composition, and timing of blood sampling.

The present findings were partly consistent with previous reports but also revealed important differences. In the baseline analysis, median NLR, PLR, and SII values were descriptively higher in the UC group than in the non-UC group, suggesting a tendency towards greater systemic inflammatory activity among patients with UC. However, the blood-based inflammatory model comprising NLR and SII showed limited discrimination, with an AUC of 0.594. These indices alone therefore provided insufficient information to distinguish UC from other inflammatory or ulcerative intestinal conditions in this cohort. In Model 1, each 1,000-unit increase in SII was associated with higher odds of a final diagnosis of UC (OR = 1.774, *P* = 0.011), whereas NLR was not statistically significant. This finding may partly reflect the strong correlation and mathematical coupling between the two indices, because NLR forms part of the SII calculation. It may also relate to the clinical spectrum of the cohort and, importantly, to the use of patients with other inflammatory or ulcerative intestinal conditions, rather than healthy individuals, as the comparator group.

These differences may reflect the distinct clinical questions addressed. Most previous studies evaluated disease activity in patients with established UC or compared active disease with remission. In contrast, the present study examined the differentiation of UC from other inflammatory or ulcerative intestinal diseases. The comparator group therefore consisted of clinically relevant patients with similar endoscopic abnormalities or inflammatory features rather than healthy individuals. Substantial overlap in systemic inflammatory indices such as NLR and SII would be expected between the two groups, limiting their discriminatory ability when used alone. These findings suggest that peripheral blood-derived inflammatory indices are better regarded as complementary to endoscopic morphological information than as the principal basis for differentiating UC. Despite the correlation between NLR and SII, the model containing both indices had slightly lower apparent AIC and Brier scores than Model 2 plus SII. However, its AUC was not significantly higher (0.739 vs. 0.731; paired DeLong *P* = 0.480), leaving the incremental discriminatory value of NLR uncertain.

### 4.4 Diagnostic performance of the combined model

Several prediction models have been developed to estimate endoscopic activity in UC. For example, Guan et al. developed a risk-prediction model for endoscopic activity and presented it as a nomogram to facilitate clinical interpretation (37). Such models have generally been developed using relatively complete biomarker data, standardized endoscopic scores, or clearly defined disease-activity categories, and some have shown high discriminatory performance. In contrast, the present study addressed the differentiation of UC from other inflammatory or ulcerative intestinal conditions in a real-world setting characterized by limited FC documentation, incomplete recording of several inflammatory markers, and variation in endoscopic reporting. Accordingly, the emphasis was on assessing the predictive contribution of routinely available endoscopic features and complete blood count-derived indices under these practical constraints.

Model 1 achieved an AUC of 0.739, indicating moderate discrimination. Its practical advantage is that its predictors can be obtained from routine endoscopy reports and complete blood count data without additional specialized biomarker testing. Compared with Model 2, adding NLR and SII produced only a modest increase in AUC. Although the paired DeLong test and exploratory continuous NRI and IDI analyses suggested some incremental predictive information, this finding was derived from the development cohort and requires confirmation in independent data. Model 1 had a slightly lower AUC than Model 4, but the difference was not statistically significant (*P* = 0.521). Because Model 4 was based on dichotomized UCEIS components rather than the total UCEIS score, this result should not be interpreted as a direct comparison between Model 1 and standard UCEIS assessment. Model 1 should therefore be regarded as a pragmatic risk-assessment tool rather than a replacement for formal UCEIS assessment or comprehensive endoscopic evaluation.

At the data-derived optimal cutoff, Model 1 had a sensitivity of 0.757, specificity of 0.648, negative predictive value of 0.901, and positive predictive value of 0.386. This performance profile supports its potential use for preliminary risk stratification and as an adjunct to clinical assessment rather than as a stand-alone test for confirming or excluding UC. Within the study population and its observed UC prevalence, a low predicted risk indicated a lower probability of UC but was insufficient to rule out the disease, whereas a high predicted risk still required further assessment incorporating histopathology, evaluation for infectious causes, FC testing, and follow-up information. For clinical interpretation, the DCA thresholds may be viewed as hypothetical predicted UC risk levels for prioritizing this further assessment after colonoscopy. The model may therefore provide an initial estimate of UC risk when colonoscopy has identified inflammatory or ulcerative lesions but other diagnostic information remains incomplete.

### 4.5 Exploratory variable selection and sensitivity analyses

In the exploratory analyses, complete-case LASSO logistic regression selected abnormal vascular pattern, bleeding, and mucosal granularity, whereas the multiple-imputation sensitivity analysis selected abnormal vascular pattern, mucosal granularity, platelet count, and bleeding. NLR and SII were not consistently selected across the different analytical strategies. When a broader set of candidate predictors was evaluated simultaneously, the predictive information provided by these blood-derived inflammatory indices may therefore have been limited or may have overlapped with that captured by endoscopic morphological features.

These findings were broadly consistent with the prespecified model comparisons, in which endoscopic morphological features appeared to provide the principal discriminatory information, whereas NLR and SII made a more limited complementary contribution. The LASSO analyses were data-driven and exploratory; they were conducted to examine the consistency of variable selection under alternative analytical and missing-data strategies rather than to replace the primary conclusions derived from the prespecified models. Given the limited number of UC cases, variable selection may be unstable, and the selected predictors should not be regarded as a definitive predictor set.

### 4.6 Strengths and limitations

A strength of this study was the use of multicenter real-world data to evaluate a diagnostic prediction model for UC while characterizing the availability and missingness of key inflammatory biomarkers, including FC, ESR, and CRP, in routine clinical records. Bootstrap resampling was used to quantify optimism, and leave-one-center-out internal-external cross-validation was used to explore transportability across the participating centers. The prespecified model comparisons were also clearly distinguished from the exploratory variable-selection and multiple-imputation analyses.

Several limitations should be acknowledged. First, the retrospective design was susceptible to selection and information bias, and endoscopy reporting, histopathological assessment, and laboratory testing practices may have differed across centers. Because endoscopic information contributed to the final clinical diagnosis and some of the same features were included as predictors, incorporation bias may have inflated the apparent effects of endoscopic variables and overall model performance. Second, substantial missingness in FC, ESR, and CRP limited their inclusion as candidate predictors, while missing blood-count data may have introduced selection bias into the complete-case analysis. The multiple-imputation analyses depended on assumptions about the missing-data mechanism and imputation models. Third, despite bootstrap validation and leave-one-center-out internal-external cross-validation, fully independent external validation has not been performed. The small number of UC cases in some held-out centers also reduced the precision of center-specific estimates. The preliminary selection of granularity and edema was not repeated within bootstrap resampling, so residual selection-related optimism cannot be excluded. Fourth, endoscopic features such as mucosal granularity are subject to observer interpretation, and centralized image review and formal assessment of interobserver agreement were unavailable. Finally, the optimal cutoff was derived from the development data and may perform less well in other populations. The model therefore requires validation in independent prospective cohorts before clinical implementation,particularly among previously undiagnosed patients undergoing an initial evaluation for suspected UC.

### 4.7 Future research directions

Future research should address three priorities. First, prospective multicenter studies should standardize the collection of FC, CRP, ESR, complete blood count parameters, and key endoscopic features to reduce the effect of missing data on study findings. Second, independent external validation should be conducted in populations from different geographical regions, levels of care, and broader disease spectra to evaluate the model's discrimination, calibration, and clinical utility. Third, simplified endoscopic features such as mucosal granularity require standardized definitions, reference image atlases, and structured training. Their reproducibility should be assessed through independent blinded evaluation by multiple endoscopists and interobserver agreement analysis using kappa statistics before they can be considered standardized adjunctive descriptors in routine endoscopy reporting.

## 5 Conclusion

In this multicenter retrospective cohort, FC was documented in only a small proportion of patients and could not be included in the primary modelling analysis. The diagnostic prediction model combining mucosal granularity, mucosal edema, NLR, and SII showed moderate discrimination and a modest increase in AUC over the two-feature endoscopic model, while its AUC did not differ significantly from that of the three-feature endoscopic comparator. The model may support preliminary UC risk stratification among patients with inflammatory or ulcerative lesions identified at colonoscopy when histopathology or FC results are not yet available. It should not be used as a stand-alone diagnostic tool or replace the standard diagnostic evaluation. Independent prospective validation with standardized endoscopic assessment and more complete biomarker collection is required before clinical implementation.

## Data Availability

The datasets presented in this article are not readily available because the dataset contains sensitive clinical information and cannot be made publicly available. Requests to access the datasets should be directed to Yunlong Sun 1299461106@qq.com.
