# Supplementary material for: Diagnostic prediction of ulcerative colitis using simplified endoscopic features and blood count-derived indices: a multicenter study
Source: Front Med (Lausanne). 2026 Oct 26;13:1991253. doi: 10.3389/fmed.2026.1991253 (PMC13745218; doi:10.3389/fmed.2026.1991253)
Supplement: Supplementary file 1 [file Table1.docx]

**Supplementary Material**

**Supplementary Figure S1. Selection of the LASSO penalty parameter by ten-fold cross-validation.**

LASSO, least absolute shrinkage and selection operator.

**Supplementary Figure S2. Predictors selected by LASSO in the complete-case analysis.**

Predictors retained by exploratory complete-case LASSO logistic regression. The analysis included 429 patients, of whom 101 had a final diagnosis of ulcerative colitis. LASSO, least absolute shrinkage and selection operator.

**Supplementary Figure S3. Adjusted associations of LASSO-selected predictors with ulcerative colitis.**

CI, confidence interval; LASSO, least absolute shrinkage and selection operator; OR, odds ratio.

**Supplementary Figure S4. Exploratory complete-case and multiple-imputation sensitivity receiver operating characteristic curves.**

Apparent receiver operating characteristic curves from the exploratory complete-case and multiple-imputation sensitivity analyses. The complete-case analysis included 429 patients, of whom 101 had ulcerative colitis, and retained vascular pattern abnormality, bleeding, and mucosal granularity. The multiple-imputation analysis included all 655 patients, of whom 135 had ulcerative colitis. AUC, area under the receiver operating characteristic curve; MI, multiple imputation; ROC, receiver operating characteristic; UC, ulcerative colitis.
